# The neglected association between central obesity markers and abdominal aortic aneurysm presence: A systematic review and meta-analysis

**DOI:** 10.3389/fcvm.2023.1044560

**Published:** 2023-02-09

**Authors:** Chang Sheng, Tinghua Liu, Shen Chen, Mingmei Liao, Pu Yang

**Affiliations:** ^1^Department of Vascular Surgery, Xiangya Hospital, Central South University, Changsha, Hunan, China; ^2^National Clinical Research Center for Geriatric Disorders, Xiangya Hospital, Central South University, Changsha, Hunan, China; ^3^Key Laboratory of Nanobiological Technology of Chinese Ministry of Health, Xiangya Hospital, Central South University, Changsha, Hunan, China

**Keywords:** abdominal aortic aneurysm, central obesity, risk factors, waist-to-hip ratio, waist circumference

## Abstract

**Purpose:**

To review the association between central obesity and abdominal aortic aneurysm (AAA).

**Materials and methods:**

The PubMed, Web of Sciences, Embase, The China national knowledge infrastructure (CNKI), and Cochrane Library were searched up to April 30, 2022. Researches includes investigation of the relationship between central obesity markers and AAA. Included studies must use recognized measures of central obesity, i.e., waist circumference (WC) and waist-to-hip ratio (WHR), or use imaging techniques to calculate abdominal fat distribution, such as computed tomography (CT) imaging.

**Results:**

Eleven clinical researches were identified of which eight discussed the association between physical examination and AAA, and three studies mainly focused on abdominal fat volume (AFV). Seven researches concluded that there was a positive correlation between markers of central obesity and AAA. Three studies found no significant link between markers of central obesity and AAA. One of the remaining studies reported different results for each sex. Three studies pooled in a meta-analysis identified correlation between central obesity and AAA presence (RR = 1.29; 95% confidence interval, 1.14–1.46).

**Conclusion:**

Central obesity plays a role in the risk of AAA. Standardized central obesity markers may be predictors of AAA. However, there was no association between abdominal fat volume and AAA. Additional relevant evidence and specific mechanisms warrant further study.

**Systematic review registration:**

https://www.crd.york.ac.uk/prospero/display_record.php?IDCRD42022332519, identifier CRD42022332519.

## 1. Introduction

Abdominal aortic aneurysm (AAA), a pathological dilatation that exceeds 1.5 times the diameter of the normal aorta, with a high mortality rate when vascular rupture occurs. Risk factors for abdominal aortic aneurysm include hypertension, being male, smoking, coronary artery disease, having a family history of abdominal aortic aneurysm, being peripheral arterial disease and older than 65 years (1). AAA rupture is associated with the excessive accumulation of fat cells in the arterial wall (2). These adipocytes are derived from perivascular adipose tissue (PVAT), a special adipose tissue reservoir distributed around the blood vessels and has been closely linked with AAA progression (3). PVAT impairs inflammation and AAA formation in animals (4). In addition, the infiltration of PVAT by macrophages and the production of some cytokines increase the formation of AAA (4). Visceral adiposity has larger biological significance for cardiovascular disease than fat from other parts of the body (5), and is also strongly associated with cardiometabolic risk (6). Over the past 40 years, the number of central obesity Chinese men and women has increased (7). Obesity can be divided into three types: general, central, and visceral. Body mass index (BMI) has been used as an indicator of obesity in many studies investigating the association between obesity and AAA, but it is not a good proxy for abdominal fat, whereas waist circumference (WC) and waist-to-hip ratios (WHR) are indices of intra-abdominal fat mass. WC can substantially improve the estimation of and corresponds well to visceral adipose tissue (VAT) (8, 9). PVAT is related to WC, WHR, and abdominal fat volume (AFV) (10).

Visceral adipose tissue (VAT) includes visceral and subcutaneous fat. Despite patients being exposed to radiation, computed tomography (CT) is a precise measure that can quantify subcutaneous and visceral fat separately (11). Central obesity refers to an imbalance between a high-energy diet and low physical activity, resulting in the accumulation of abdominal fat. Abdominal fat can secretes inflammatory cytokines and the adjacent action of PVAT can lead to abdominal aortic disease (12). Thus, this study hypothesizes that visceral obesity plays a more prominent role in the formation of AAA, and aimed to review the precise association between AAA and central obesity by considering central obesity represented by WC, WHR, and abdominal fat volume (AFV) as measurement indices, including the possible rationale for the occurrence of AAA due to obesity.

## 2. Materials and methods

### 2.1. Protocol

This review followed the Preferred Reporting Items for Systematic Reviews and Meta-Analyses (PRISMA) guidelines. This study focused on studies that exploring the association between markers of AAA presence and central obesity (PROSPERO CRD42022332519).

### 2.2. Search strategy

Systematic literature searches were performed using PubMed, Web of Sciences, Embase, CNKI, and the Cochrane Library up to April 30, 2022. The PubMed was searched using the following terms: [“obesity” OR “waist circumference (WC)” OR “waist-to-hip ratio (WHR)” OR “body mass index” OR “body adipose distribution”] AND (“aortic aneurysm,” “abdominal”). We retrieved the Cochrane Library, and CNKI found no filters available. One author developed the search strategy making any necessary modifications to the search terms. Furthermore, search the reference list in the relevant results to increase the number of valid documents.

### 2.3. Eligibility requirements

The eligible studies should use a recognized symbol of central obesity, namely WHR, WC, waist-to-height ratio, or utilize medical imaging tools to quantify AFV. No restrictions were added to the type of publications included or to the study size. No language limitations were applied. The detection method of AAA in the target study is reliable. Researches were rejected if AAA was not well-defined or pertinent results of markers of central obesity were not reported (Table 1).

**TABLE 1:** Central obesity definitions were used in each study.

References	Markers of central obesity	The nature of the evaluation method
Lederle et al., (22)	WC	Subjective (Participant reported)
Jamrozik et al., (23)	WHR	Subjective (Participant reported)
Lederle et al., (24)	WC	Subjective (Participant reported)
Singh et al., (25)	WHR	Subjective (Participant reported)
Golledge et al., (18)	WHR, WC	Objective (Assessor measured)
Stackelberg et al., (19)	WC	Subjective (Participant reported)
Fattahi et al., (20)	WC	Objective (Assessor measured)
Stackelberg et al., (21)	WC	Subjective (Participant reported)
Apoloni et al., (29)	AC, AFV	Objective (CT)
Dias-Neto et al., (30)	AFV	Objective (CT)
Cronin et al., (31)	AFV	Objective (CT)

WHR, waist-to-hip ratio; WC, waist circumference; AFV, abdominal fat volume; CT, computed tomography; AC, abdominal circumference.

### 2.4. Data abstraction

For the final obtained studies, two authors independently took data. Data extracted from qualified Publications included AAA presence, central obesity assessment measure, study design, country, population, age, sex, hypertension, smoking history, diabetes mellitus, imaging method, time-interval for follow-up (if reported), type of statistical analysis, odds ratio (OR), *P* value, 95% confidence interval (95% CI) and author’s findings, multivariate or univariate analysis results. Finally, the disagreement was resolved through a discussion between authors.

### 2.5. Quality assessment

ROBINS-I (a tool for assessing the risk of bias in non-randomized studies) (13) was used to assess the performance and heterogeneity of the included researches. This tool has seven indicators, the first two domains evaluate confusion and selection of study participants, and the third domain is the classification of the intervention. The other four domains assess problems after intervention initiation, including missing data, bias due to differences from the intended intervention, choice of reported outcomes and outcome measures (13). We considered “central obesity” as an exposure. The purpose of this study was to provide overall quality assessment results. The results of each assessment were compared between authors, and divergences were resolved by negotiation.

### 2.6. Statistical analysis

Stata 14.0 software (version 14.0) was used for data analysis. Statistical heterogeneity was evaluated using *I*^2^ statistic tests and Cochran’s Q. When significant heterogeneity (PQ > 0.10, *I*^2^ > 50%) was calculated to obtain, the random-effects model was carried out. We performed sensitivity analyses (omitting one study at a time) to assess the stability of the results.

## 3. Results

### 3.1. Search results

**FIGURE 1:**
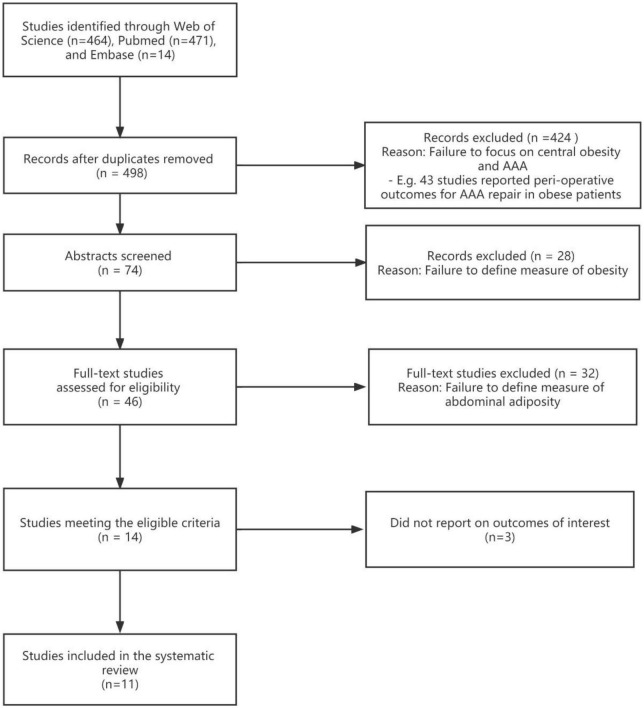
Flow diagram to illustrate the trials identified for this review.

At first, a total of 949 published studies were identified, including 464 studies found on the Web of Science and 471 from PubMed; Embase retrieved 14 studies, which were excluded (Figure 1). One meta-analysis, focusing on the relationship between BMI and the existence of AAA (14). Duplicate studies were removed and 498 independent publications were included, all identified publications were written in English. A total of 424 studies were excluded due to failure to focus on AAA and content discrepancies such as a focus on perioperative outcomes in obese patients with AAA. Of the 74 studies that evaluated abstracts, 28 were excluded because they failed to define obesity measures. A full-text review of the literature was then conducted and 36 of these were excluded. Twenty studies were excluded because they failed to define a measure of abdominal obesity. Three studies were excluded because they investigated the association between markers of central obesity and aortic dimensions; AAA was not well-defined (15–17). Finally, 11 studies were selected of which eight discussed the association between physical examination (WC or WHR) and AAA, and three studies mainly focused on AFV (Figure 1).

### 3.2. Characteristics of included studies

The eight studies focused on the relationship between physical examination findings and AAA presence had sample sizes ranging from 5,000 to 80,000 subjects (12203, 63655, 5817, 14249, 52745, 11745, 73451, 6386) (18–25). Four study groups included only men (18, 20, 21, 23), and the rest included both men and women (19, 22, 24, 25). Six studies with asymptomatic subjects from the community; the rest two study populations were from America’s medical institutions, and there was an inclusive relationship between them (22, 24). Three studies were conducted in Sweden (19–21), two in Australia (18, 23), and one in Norway (25). Two studies (19, 21) were based on the cohort of Swedish men (COSM) (26), for which the leading author was the same for both studies (Tables 2, 3).

**TABLE 2:** Summary of studies assessing the association of central obesity with abdominal aortic aneurysm (AAA) presence.

References	Subject groups (n)	Male n (%)	Mean age (years)	Ever smoked n (%)	Hypertension n (%)
**WHR or WC reporting studies**					
Lederle et al., (22)	No AAA (50215)	51374 (97.4)	65.6 ± 8.0	38979 (73.9)	27744 (52.6)
	AAA (2530)				
Jamrozik et al., (23)	No AAA (10872)	10872 (100)	65–69^a^	7589 (69.8)	4251 (39.1)
	AAA (873)	873 (100)	70–74^a^	756 (86.6)	434 (49.7)
Lederle et al., (24)	No AAA (70085)	71394 (97.2)	66.2 ± 7.1	55456 (75.5)	40472 (55.1)
	AAA (3366)				
Singh et al., (25)	No AAA (6049)	2699 (44.6)	Men: 60.8 ± 10.0	1320 (48.9)	470 (17.4)
			Women: 61.2 ± 10.2	871 (26.0)	603 (18.0)
	AAA (337)	263 (78.0)	Men: 66.4 ± 6.1	110 (41.8)	77 (29.1)
			Women: 69.4 ± 5.4	13 (17.4)	27 (36.8)
Golledge et al., (18)	No AAA (11328)	11328 (100)	72 ± 4	7880 (70)	4823 (43)
	AAA (875)	875 (100)	74 ± 4	758 (87)	488 (56)
Stackelberg et al., (19)	No AAA (63058)	33504 (53.1)			
	AAA (597)	492 (82.4)			
Fattahi et al., (20)	NO AAA (5664)	5664 (100)	50.1 ± 0.4	3635 (65.2)^b^	134.0 ± 16.9^c^
	AAA (153)	153 (100)	50.1 ± 0.5	140 (91.5)	136.7 ± 15.9^c^
Stackelberg et al., (21)	NO AAA (14081)	14081 (100)	55.3 ± 4.2	(21.8)^d^	(4.6)
	AAA (168)	168 (100)	56.7 ± 4.3	(64.9)^d^	(6.3)
Apoloni et al., (29)	NO AAA (180)	(49.4)	65 ± 7		
	AAA (268)	(79.2)	69 ± 8		
Dias-neto et al., (30)	NO AAA (97)	50 (51.5)	64.26 ± 11.36		56 (60.2)^b^
	AAA (140)	113 (80.7)	71.57 ± 8.21		104 (79.4)^b^
Cronin et al., (31)	NO AAA (181)	125 (69)	66^e^	146 (81)	131 (72)
	AAA (196)	157 (80)	73^e^	172 (88)	156 (80)

^a^The age group with the highest percentage.

^b^The study exists missing data.

^c^Mean systolic blood pressure (mmHg).

^d^Current smokers %.

^e^Standard deviation was not reported.

**TABLE 3:** Summary of studies assessing the association of central obesity with abdominal aortic aneurysm (AAA) presence.

References	Country	Population	Markers of central obesity	OR (95% CI) (univariate)	OR (95% CI) (multivariable)
**WHR or WC reporting studies**					
Lederle et al., (22)	America	Hospital	WC per 11 cm	OR not reported	AAA of 3.0 to 3.9 cm vs. Normal: 1.02 (0.92–1.13)
					AAA of at least 4.0 cm vs. Normal: 1.19 (1.03–1.38)
Jamrozik et al., (23)	Australia	Community	WHR > 0.9	OR not reported	1.4 (1.2–1.8)^a^
Lederle et al., (24)	America	Hospital	WC per 11 cm	OR not reported	AAA of 3.0 to 3.9 cm vs. Normal: 1.06 (0.98–1.14)
					AAA of at least 4.0 cm vs. Normal: 1.15 (1.03–1.29)
Singh et al., (25)	Norway	Community	WHR per 0.1	OR not reported	Men: 1.12 (0.86–1.44)
					Women: 1.48 (1.04–2.10)*
Golledge et al., (18)	Australia	Community	WHR per 0.1^b^	OR not reported	1.22 (1.09–1.37)*
			WC per 10 cm^b^	OR not reported	1.14 (1.06–1.22)*
Stackelberg et al., (19)	Sweden	Community	WC: 94–101.9 cm	OR not reported	1⋅30 (1⋅05, 1⋅60)^c^
			WC ≥ 102 cm	OR not reported	1⋅26 (0⋅97, 1⋅62)^c^
Fattahi et al., (20)	Sweden	Community	WC cm	1.031 (1.014–1.048)*^d^	1.019 (1.002–1.037)*^e^
Stackelberg et al., (21)	Sweden	Community	WC ≥ 94 cm	1.40 (0.98–2.00)^d^	0.94 (0.61–1.44)^e^
AFV reporting studies					
Apoloni et al., (29)	Brazil	Hospital	WC	OR not reported	OR not reported
			AFV	OR not reported	OR not reported
Dias-neto et al., (30)	Portugal	Hospital	VAT/SAT ratio	0.254 (0.100)*^f^	−0.035 (0.147)^g^
			PVAT density	−2.434 (4.301)^f^	2.605 (9.630)^g^
			Intra-individual PVAT differences	12.869 (3.645)*^f^	13.051 (4.625)*^g^
Cronin et al., (31)	Australia	Hospital	Visceral to total abdominal adipose volume ratio	OR not reported	1.625 (0.713–3.703)^h^

*P-value is less than 0.05 (*P*-values were not reported in some studies).

^a^Age-adjusted Odds ratio.

^b^Approximately 1 SD.

^c^Multivariable adjusted relative risk.

^d^Univariate HR (95% CI).

^e^Multivariate HR (95% CI).

^f^Unadjusted β(standard error, SE), β regression.

^g^Risk factors and cardiovascular diseases adjusted β(standard error, SE), β regression.

^h^OR of adipose volume ratio Quartile 4 vs. Quartile 1.

The biggest difference between studies was the variation in the definition and measurement of obesity (Table 3). Combined analysis is difficult to perform as some studies used classified variables to define obesity, whereas others used continuous variables. Two studies obtained WC or WHR by assessor measurements, and the remaining six studies by participant reports (18, 20, 22–25). Three studies grouped WC and WHR according to the World Health Organization classification (19, 21, 27). However, their grouping was not entirely standardized. Commonly used markers of central obesity are WC ≥ 88 cm in women and ≥ 102 cm in men; and WHR > 0.8 in women and > 1.0 in men (28).

Three studies mainly focused on AFV. Their sample sizes ranged from 200 to 500 subjects (448, 237, 377) (29–31). The patients in these studies were from medical institutions, such as vascular clinics. These studies were conducted in Brazil (29), Portugal (30), and Australia (31) respectively. There was a marked variation in the AFV (Table 3). Apoloni et al. (29) positioned CT images of abdominal obesity at the level of the third and fourth lumbar vertebrae, obtaining visceral fat area, subcutaneous fat area (SFA), visceral/subcutaneous ratio and WC, as validated by Irlbeck et al. (32). Dias-neto et al. (30) studied subcutaneous and visceral adipose tissue in a designated CT slice (the axial slice was 60 mm cranial to the L4/L5 intervertebral disk), as shown by Demerath et al. (33). Moreover, measurements [region of interest (ROI) at each level is a 10-mm area surrounding the outer contour of the aorta] were performed using the method detailed by Schlett et al. (10). In the study of Cronin et al. (31), through axial CTA images processing, the subcutaneous adipose volume, total adipose volume and visceral-to-AFV ratio can be obtained (Table 3). Apoloni et al. also used WC as a measurement Method of central obesity (29).

### 3.3. Research quality

Six studies (18, 20–22, 24, 25) were judged to be at a moderate risk of bias due to confounding factors. Five studies (19–22, 24) were at moderate risk of bias as missing data. One study excluded women because of the low prevalence of AAA, and this study was judged to have moderate selection bias (23). All remaining parts of the study were judged to be at low risk of bias (Table 4). In addition, heterogeneity arises from differences in central obesity markers.

**TABLE 4:** Risk of bias assessment for observational studies (Robins-I tool).

References	Confounding	Selection	Measurement of interventions	Deviations from intended interventions	Missing data	Measurement of outcomes	Selection of the reported result
Lederle et al., (22)	moderate	low	low	low	moderate	low	low
Jamrozik et al., (23)	low	moderate	low	low	low	low	low
Lederle et al., (24)	moderate	low	low	low	moderate	low	low
Singh et al., (25)	moderate	low	low	low	low	low	low
Golledg et al., (18)	moderate	low	low	low	low	low	low
Stackelberg et al., (19)	low	low	low	low	moderate	low	low
Fattahi et al., (20)	moderate	low	low	low	moderate	low	low
Stackelberg et al., (21)	moderate	low	low	low	moderate	low	low
Apoloni et al., (29)	low	low	low	low	low	low	low
Dias-neto et al., (30)	low	low	low	low	low	low	low
Cronin et al., (31)	low	low	low	low	low	low	low

### 3.4. The association between central obesity markers with AAA presence

The positive association between central obesity and the presence of AAA was studied in seven (Table 3; 18–20, 22–25), when confounding factors were considered, five of the studies were at moderate risk. In an updated study by Lederle et al. (22), a combined group indicated that AAA of at least 4.0 cm was significantly associated for every 11 cm WC increase (OR, 1.16; 95% CI, 1.07–1.27). Both studies found that WHR was positively related to the risk of AAA (23, 25). In a study by Golledge et al. (18) when WC was used as a measure of obesity, a total of 4,714 men (39%) had obesity at the start and a total of 386 had AAA (OR, 1.28; 95% CI, 1.11–1.47). In addition, every 0.1 WHR (OR, 1.22; 95% CI, 1.09–1.37) and every 10 cm WC (OR, 1.14; 95% CI, 1.06–1.22) were positively associated with AAA, and this association was more significant for larger AAA (18). An observational cohort study was conducted by Stackelberg et al. (19). Data were obtained from two large cohorts (Swedish Mammography Cohort and Swedish Male Cohort) of 63,655 individuals between the ages of 46–84 years. Multivariate analysis showed that those with increased waist circumference had a 30% higher risk of developing AAA than those without (RR 1.30, 95% CI, 1.05–1.60) (19). Meanwhile, the risk of AAA increased with increasing WC (RR 1.15, 95% CI, 1.05–1.26) until > 100 cm in men and > 88 cm in women (19). Fattahi et al. conducted a point prevalence study (20), aortic ultrasound results of 5,817 men aged 50 years were compared with the same population 15 years later. In univariate regression analysis, waist circumference was associated with AAA (HR, 1.031; CI, 1.014–1.048; *P* < 0.001). Furthermore, multivariate analysis showed that rising WC was also link with the AAA development [hazard ratio (HR), 1.019; 95% CI, 1.002–1.037; *P* < 0.007] (20).

Three studies did not consider an association between AAA and central obesity (21, 30, 31). Stackelberg et al. reported a higher WC in participants with an AAA than in those without an AAA, but the results were not statistically significant (Table 3; 21). In a case-control investigation by Cronin et al. (31), the visceral-to-AFV ratio was not statistically associated with AAA after adjusting for other risk factors. Specifically, patients with a visceral-to-AFV ratio in quartile had a nearly twofold increased risk of AAA, but the confidence interval crosses 1 (95% CI 0.71–3.70; *P* = 0.248) (31). Dias-Neto et al. (30) conducted a multicenter retrospective case-control study, patients with AAA had a higher rate of visceral obesity compared to controls. However, this association disappeared in the multivariate analysis (Table 3). They found that patients showed higher PVAT density in CT region 2 (slice of maximal aortic diameter) than in CT Region 1 (uppermost slice of infrarenal aorta) (mean difference 7.97, 95% CI 3.31–12.63; *P* = 0.001). In addition, the presence of AAA increased intraindividual PVAT differences by 13.2 units (CT values) (Table 3), the difference between the average PVAT in region 2 minus the average PVAT in region 1 is equal to the intra-individual PVAT differences.

A cross-sectional study was conducted by Apoloni et al. (29). Among males, AAA patients were similar to controls in terms of obesity. The difference in experience is surprising: in 339 men with AAA, there was a negative association between VAT and aortic diameter. Unlike the control group, the AAA group had older women and also observed lower WC and SFA.

### 3.5. Meta-analysis and sensitivity analysis

**FIGURE 2:**
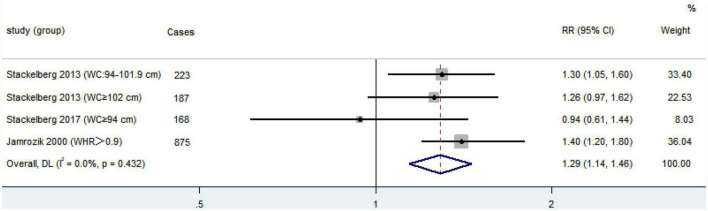
Forest plot showing adjusted estimates (*RR*) and 95% CIs for the association between central obesity and abdominal aortic aneurysm presence in case-control and cohort studies using a random effects model. Gray squares and horizontal lines represent study-specific estimates and 95% CI. The size of the square indicates the study weight. Diamonds are pooled estimates (center) and 95% confidence intervals (width) using a random effects analysis. *I*^2^, estimate for the proportion of variability between studies that is due to inter-study heterogeneity; *P*-value was calculated by Chi-square test of the Cochrane *Q* statistic. *RR*, relative risk; CI, confidence interval.

Three studies grouped obesity indicators based on central obesity criteria (19, 21, 23). One study regrouped people according to the degree of central obesity, and the two groups came to different conclusions (19). In this study, a cumulative meta-analysis of research themes was conducted. We used a fixed-effect model (*I*^2^ = 0.0%, *P* = 0.432), and pooled RRs to identify a correlation between central obesity and AAA in the present meta-analysis (RR = 1.29; 95% CI, 1.14–1.46; Figure 2). A sensitivity analysis was achieved by excluding individual studies at a time to test the effect of individual data on the pooled RRs, and the results showed that no individual study belly reversed the rest of the study (Table 5).

**TABLE 5:** Sensitivity analysis about central obesity and abdominal aortic aneurysm presence of the meta-analysis.

Study omitted	RR	95% CI	*I* ^2^	*P*-value
Stackelberg et al., (19)	1.29	1.11–1.49	27.1%	0.254
Stackelberg et al., (19)	1.30	1.13–1.49	26.0%	0.259
Stackelberg et al., (21)	1.33	1.17–1.51	0.0%	0.794
Jamrozik et al., (23)	1.23	1.06–1.44	0.0%	0.406

## 4. Discussion

This systematic evaluation found that the included studies reported a positive association of WC or WHR with AAA and showed that AFV was not associated with AAA present. In the quantitative analysis, we considered obesity as a risk factor for AAA. However, confounding factors existed in most studies, with some collinearity effects. In all studies, obese patients were more likely to have comorbidities and higher detection rates of AAA. This may have exaggerated the association between obesity and AAA. Surprisingly, two studies based on COSM with the same author (19, 21). Reported conflicting results. In the former, the bigger sample size gives more confidence that the sample is representative (19). The latter was published 4 years later and used statistical methods that took time into account. Commonly used markers of central obesity are WC ≥ 88 cm in women and ≥ 102 cm in men; and WHR > 0.8 in women and > 1.0 in men (28). The WC grouping of the two studies may have diluted the association between WC and AAA. Questionnaire recovery was used as the WC collection method in these two studies.

Dias-Neto et al. and colleagues (30) considered the existence of AAA as an independent determinant of higher PVAT density in the aneurysmal sac and less significantly in the aneurysmal neck. They also found that intraindividual PVAT differences were positively correlated with aneurysmal sac volume and suggested that the local mechanism of action of PVAT promotes AAA pathophysiology. Further studies with larger sample sizes are required to establish this link. In contrast, Apoloni et al. (29) demonstrated a negative correlation between VAT and abdominal aortic diameter in men. However, further research is required to confirm this. Artificial intelligence is an attractive decision-making tool, and its research on AAA has received increasing attention (34). Its methodology can take into account a large number of features that are not limited to the volume of fat, but will also include the morphology of the fat tissue, the texture of the fat tissue, etc., (11). In the future, artificial intelligence methods based on imaging data may be required to investigate regional fat density and to further study the relationship between obesity and AAA.

Measurements followed the standards of International Society for the Advancement of Kinanthropometry (35). In this study by Fattahi et al., participants were clinically examined by primary care nurses who recorded their WC (20). This standardized approach to data collection provided confidence in the accuracy of the measurements.

Three studies employed CT, but used different criteria for the quantitative analysis of abdominal fat (29–31). As mentioned above, we hypothesize that a relationship between central obesity and AAA is probable. However, the relationship between central obesity and AAA should be determined in further studies using normalized AFV, and threshold ranges defined from adipose tissue. In addition, the development of CT technology will enhance research related to the assessment or quantification of perivascular adipose tissue.

Larger individuals may naturally have larger aortic diameters. Moreover, in one of the excluded studies, found that increased sagittal abdominal diameter did not increase the incidence of AAA (36). Other possible explanations are that visceral obesity is not a risk factor for AAA progression, but rather a phenotype that represents an imbalance in energy intake. In this perspective, the drivers of AAA may be overeating and less exercise, which can lead to abdominal obesity. Furthermore, there is a potential role for different management of patients depending on their BMI.

Nordkvist et al. showed that a high intake of vegetables and fruits decreases the risk of incident AAA (37). High-fat diets, smoking, and pollution are responsible for the development of adipose tissue into a reservoir of lipophilic toxic compounds (38). This is important because organic pollutants are lipophilic, bioaccumulative and persistent in the food chain. Human intake of high-fat foods is the primary route of exposure to these toxic substances, which also slowly accumulate in human fatty tissue. Animal studies have shown that increased persistent organic pollutant concentrations in visceral fat and perianeurysmal fat contribute to the incidence and growth of AAA (39). Specifically, polychlorinated biphenyls (PCBs) are a commercially produced environmental contaminant to which humans are exposed through food, water, and inhalation. PCBs are capable of increasing macrophage recruitment within the epicardium of the abdominal aorta and enhancing AAA formation (39). Systematic reviews of prospective studies have found that AAA risk can be reduced by higher physical activity; however, further studies are required to clarify the dose-response relationship between different sub-types and AAA risk (40). One study showed that patients who underwent bariatric surgery had restored vasodilatory capacity in PVAT and reduced oxidative stress and perivascular inflammation (41).

Previous studie have shown a negative association between diabetes and AAA, and this is when the positive association between obesity and AAA becomes interesting (24). A large trial screening for AAA found an inverse association between aortic diameter and blood glucose in men without diabetes (42). Thus, we hypothesize that insulin resistance, which is usually prevalent in obese subjects, is unlikely to be the cause of the association between obesity and AAA. Additionally, obese individuals may have enhanced muscle strength, which is associated with better prognosis (43, 44).

All of these studies are influenced by many confounding factors and are difficult to eliminate, such as undiagnosed chronic diseases, smoking history. Despite the obesity paradox, current data do not support an increase in AFV in patients with AAA. In addition to energy storage, adipose tissue also influences glucose metabolism and vascular biology (12).

## 5. Conclusion

The association between central obesity and AAA is positive, there is some heterogeneity in this study regarding the different definitions of central obesity, a more comprehensive exploration is needed, and it will be necessary to use standard obesity measurement methods to evaluate this relationship in the future. We hope to identify the factors related to AAA and provide evidence for AAA prevention.

## Data availability statement

The original contributions presented in this study are included in this article/supplementary material, further inquiries can be directed to the corresponding authors.

## Author contributions

CS: writing – initial draft, methodology, search the database, formal analysis, and writing – original draft and manuscript. TL: writing – initial draft and search the database. CS and TL review the researches, and collect the data. SC: writing – initial draft. ML and PY: supervision and writing – initial draft, review, and editing. All authors contributed to the article and approved the submitted version.

## References

[1] Haque K Bhargava P. Abdominal aortic aneurysm. *Am Fam Physician.* (2022) 106:165–72. 35977132

[2] Kugo H Zaima N Tanaka H Mouri Y Yanagimoto K Hayamizu K Adipocyte in vascular wall can induce the rupture of abdominal aortic aneurysm. *Sci Rep.* (2016) 6:31268. 10.1038/srep31268 27499372 PMC4976321

[3] Kugo H Zaima N Tanaka H Hashimoto K Miyamoto C Sawaragi A Pathological analysis of the ruptured vascular wall of hypoperfusion-induced abdominal aortic aneurysm animal model. *J Oleo Sci.* (2017) 66:499–506. 10.5650/jos.ess16219 28381776

[4] Police S Thatcher S Charnigo R Daugherty A Cassis L. Obesity promotes inflammation in periaortic adipose tissue and angiotensin II-induced abdominal aortic aneurysm formation. *Arterioscler Thromb Vasc Biol.* (2009) 29:1458–64. 10.1161/ATVBAHA.109.192658 19608970 PMC2753598

[5] Matsuzawa Y. Establishment of a concept of visceral fat syndrome and discovery of adiponectin. *Proc Jpn Acad Ser B Phys Biol Sci.* (2010) 86:131–41. 10.2183/pjab.86.131 20154470 PMC3417563

[6] Dutheil F Gordon B Naughton G Crendal E Courteix D Chaplais E Cardiovascular risk of adipokines: a review. *J Int Med Res.* (2018) 46:2082–95. 10.1177/0300060517706578 28974138 PMC6023062

[7] Pan X Wang L Pan A. Epidemiology and determinants of obesity in China. *Lancet Diabetes Endocrinol.* (2021) 6:373–92. 10.1016/S2213-8587(21)00045-0 34022156

[8] Boone S van Smeden M Rosendaal F le Cessie S Groenwold R Jukema J. Evaluation of the value of waist circumference and metabolomics in the estimation of visceral adipose tissue. *Am J Epidemiol.* (2022) 5:886–99. 10.1093/aje/kwab298 35015809 PMC9071575

[9] Bertoli S Leone A Vignati L Bedogni G Martínez-González M Bes-Rastrollo M Adherence to the Mediterranean diet is inversely associated with visceral abdominal tissue in Caucasian subjects. *Clin Nutr.* (2015) 34:1266–72. 10.1016/j.clnu.2015.10.003 26499033

[10] Schlett C Massaro J Lehman S Bamberg F O’Donnell C Fox C Novel measurements of periaortic adipose tissue in comparison to anthropometric measures of obesity, and abdominal adipose tissue. *Int J Obes.* (2009) 33:226–32. 10.1038/ijo.2008.267 19139753 PMC3779879

[11] Hu G Ding N Wang Z Jin Z. The association of body composition with abdominal aortic aneurysm growth after endovascular aneurysm repair. *Insights Imaging.* (2022) 13:76. 10.1186/s13244-022-01187-7 35467156 PMC9038972

[12] Ye T Zhang G Liu H Shi J Qiu H Liu Y Relationships between perivascular adipose tissue and abdominal aortic aneurysms. *Front Endocrinol.* (2021) 12:704845. 10.3389/fendo.2021.704845 34194399 PMC8236981

[13] Sterne J Hernán M Reeves B Savoviæ J Berkman N Viswanathan M ROBINS-I: a tool for assessing risk of bias in non-randomised studies of interventions. *BMJ.* (2016) 355:i4919. 10.1136/bmj.i4919 27733354 PMC5062054

[14] Takagi H Umemoto T. A meta-analysis of the association of obesity with abdominal aortic aneurysm presence. *Int Angiol.* (2015) 34:383–91. 24945917

[15] Gorter P Visseren F Moll F van der Graaf Y Smart Study Group. Intra-abdominal fat and metabolic syndrome are associated with larger infrarenal aortic diameters in patients with clinically evident arterial disease. *J Vasc Surg.* (2008) 48:114–20. 10.1016/j.jvs.2008.02.020 18440755

[16] Zhu F Arshi B Ikram M De Knegt R Kavousi M. Sex-specific normal values and determinants of infrarenal abdominal aortic diameter among non-aneurysmal elderly population. *Sci Rep.* (2021) 11:17762. 10.1038/s41598-021-97209-3 34493798 PMC8423780

[17] Thanassoulis G Massaro J Corsini E Rogers I Schlett C Meigs J Periaortic adipose tissue and aortic dimensions in the Framingham heart study. *J Am Heart Assoc.* (2012) 1:e000885. 10.1161/JAHA.112.000885 23316310 PMC3540669

[18] Golledge J Clancy P Jamrozik K Norman P. Obesity, adipokines, and abdominal aortic aneurysm: health in men study. *Circulation.* (2007) 116:2275–9. 10.1161/CIRCULATIONAHA.107.717926 17967974

[19] Stackelberg O Björck M Sadr-Azodi O Larsson S Orsini N Wolk A. Obesity and abdominal aortic aneurysm. *Br J Surg.* (2013) 100:360–6. 10.1002/bjs.8983 23203847

[20] Fattahi N Rosenblad A Kragsterman B Hultgren R. Risk factors in 50-year-old men predicting development of abdominal aortic aneurysm. *J Vasc Surg.* (2020) 72:1337–46.e1. 10.1016/j.jvs.2019.11.062 32115319

[21] Stackelberg O Wolk A Eliasson K Hellberg A Bersztel A Larsson S Lifestyle and risk of screening-detected abdominal aortic aneurysm in men. *J Am Heart Assoc.* (2017) 6:e004725. 10.1161/JAHA.116.004725 28490522 PMC5524061

[22] Lederle F Johnson G Wilson S Chute E Hye R Makaroun M The aneurysm detection and management study screening program: validation cohort and final results. Aneurysm detection and management veterans affairs cooperative study investigators. *Arch Intern Med.* (2000) 160:1425–30. 10.1001/archinte.160.10.1425 10826454

[23] Jamrozik K Norman P Spencer C Parsons R Tuohy R Lawrence-Brown M Screening for abdominal aortic aneurysm: lessons from a population-based study. *Med J Aust.* (2000) 173:345–50. 10.5694/j.1326-5377.2000.tb125684.x 11062788

[24] Lederle F Johnson G Wilson S Chute E Littooy F Bandyk D Prevalence and associations of abdominal aortic aneurysm detected through screening. Aneurysm detection and management (ADAM) veterans affairs cooperative study group. *Ann Intern Med.* (1997) 126:441–9. 10.7326/0003-4819-126-6-199703150-00004 9072929

[25] Singh K Bønaa K Jacobsen B Bjørk L Solberg S. Prevalence of and risk factors for abdominal aortic aneurysms in a population-based study : the Tromsø study. *Am J Epidemiol.* (2001) 154:236–44. 10.1093/aje/154.3.236 11479188

[26] Orsini N Bellocco R Bottai M Pagano M Michaelsson K Wolk A. Combined effects of obesity and physical activity in predicting mortality among men. *J Intern Med.* (2008) 264:442–51. 10.1111/j.1365-2796.2008.01985.x 18513340

[27] World Health Organization. Obesity: preventing and managing the global epidemic. Report of a WHO consultation. *World Health Organ Tech Rep Ser.* (2000) 894:1–253. 11234459

[28] Poirier P Giles T Bray G Hong Y Stern J Pi-Sunyer F Obesity and cardiovascular disease: pathophysiology, evaluation, and effect of weight loss: an update of the 1997 American heart association scientific statement on obesity and heart disease from the obesity committee of the council on nutrition, physical activity, and metabolism. *Circulation.* (2006) 113:898–918. 10.1161/CIRCULATIONAHA.106.171016 16380542

[29] Apoloni R Zerati A Wolosker N Saes G Wolosker M Curado T Analysis of the correlation between central obesity and abdominal aortic diseases. *Ann Vasc Surg.* (2019) 54:176–84. 10.1016/j.avsg.2018.06.016 30103051

[30] Dias-Neto M Meekel J van Schaik T Hoozemans J Sousa-Nunes F Henriques-Coelho T High density of periaortic adipose tissue in abdominal aortic aneurysm. *Eur J Vasc Endovasc Surg.* (2018) 56:663–71. 10.1016/j.ejvs.2018.07.008 30115505

[31] Cronin O Liu D Bradshaw B Iyer V Buttner P Cunningham M Visceral adiposity is not associated with abdominal aortic aneurysm presence and growth. *Vasc Med.* (2014) 19:272–80. 10.1177/1358863X14537883 24948557

[32] Irlbeck T Massaro J Bamberg F O’Donnell C Hoffmann U Fox C. Association between single-slice measurements of visceral and abdominal subcutaneous adipose tissue with volumetric measurements: the Framingham heart study. *Int J Obes.* (2010) 34:781–7. 10.1038/ijo.2009.279 20065971 PMC2982778

[33] Demerath E Shen W Lee M Choh A Czerwinski S Siervogel R Approximation of total visceral adipose tissue with a single magnetic resonance image. *Am J Clin Nutr.* (2007) 85:362–8. 10.1093/ajcn/85.2.362 17284730 PMC2883309

[34] Raffort J Adam C Carrier M Ballaith A Coscas R Jean-Baptiste E Artificial intelligence in abdominal aortic aneurysm. *J Vasc Surg.* (2020) 72:321–33.e1. 10.1016/j.jvs.2019.12.026 32093909

[35] Norton K Whittinghan N Carter L Kerr D Gore C Marfell-Jones M. Measurement techniques in anthropometry. In: Norton K Olds T editors. *Antropometrica.* Sydney, NSW: UNSW (1996).

[36] Iribarren C Darbinian J Go A Fireman B Lee C Grey D. Traditional and novel risk factors for clinically diagnosed abdominal aortic aneurysm: the Kaiser multiphasic health checkup cohort study. *Ann Epidemiol.* (2007) 17:669–78. 10.1016/j.annepidem.2007.02.004 17512215

[37] Nordkvist S Sonestedt E Acosta S. Adherence to diet recommendations and risk of abdominal aortic aneurysm in the Malmö diet and cancer study. *Sci Rep.* (2018) 8:2017. 10.1038/s41598-018-20415-z 29386636 PMC5792541

[38] Maeda N Shimomura I Kishida K Nishizawa H Matsuda M Nagaretani H Diet-induced insulin resistance in mice lacking adiponectin/ACRP30. *Nat Med.* (2002) 8:731–7. 10.1038/nm724 12068289

[39] Arsenescu V Arsenescu R Parulkar M Karounos M Zhang X Baker N Polychlorinated biphenyl 77 augments angiotensin II-induced atherosclerosis and abdominal aortic aneurysms in male apolipoprotein E deficient mice. *Toxicol Appl Pharmacol.* (2011) 257:148–54. 10.1016/j.taap.2011.08.028 21925196 PMC3220787

[40] Aune D Sen A Kobeissi E Hamer M Norat T Riboli E. Physical activity and the risk of abdominal aortic aneurysm: a systematic review and meta-analysis of prospective studies. *Sci Rep.* (2020) 10:22287. 10.1038/s41598-020-76306-9 33339835 PMC7749100

[41] Aghamohammadzadeh R Greenstein A Yadav R Jeziorska M Hama S Soltani F Effects of bariatric surgery on human small artery function: evidence for reduction in perivascular adipocyte inflammation, and the restoration of normal anticontractile activity despite persistent obesity. *J Am Coll Cardiol.* (2013) 2:128–35. 10.1016/j.jacc.2013.04.027 23665100 PMC3791397

[42] Le M Jamrozik K Davis T Norman P. Negative association between infra-renal aortic diameter and glycaemia: the health in men study. *Eur J Vasc Endovasc Surg.* (2007) 33:599–604. 10.1016/j.ejvs.2006.12.017 17307366

[43] Artero E Lee D Lavie C España-Romero V Sui X Church T Effects of muscular strength on cardiovascular risk factors and prognosis. *J Cardiopulm Rehabil Prev.* (2012) 32:351–8. 10.1097/HCR.0b013e3182642688 22885613 PMC3496010

[44] Artero E Lee D Ruiz J Sui X Ortega F Church T A prospective study of muscular strength and all-cause mortality in men with hypertension. *J Am Coll Cardiol.* (2011) 57:1831–7. 10.1016/j.jacc.2010.12.025 21527158 PMC3098120

